# Shared bacterial communities between soil, stored drinking water, and hands in rural Bangladeshi households

**DOI:** 10.1016/j.wroa.2020.100056

**Published:** 2020-05-23

**Authors:** Erica R. Fuhrmeister, Ayse Ercumen, Jessica A. Grembi, Mahfuza Islam, Amy J. Pickering, Kara L. Nelson

**Affiliations:** a Department of Civil and Environmental Engineering, University of California, Berkeley, CA, 94720, United States; b School of Public Health, University of California, Berkeley, CA, 94720, United States; c Department of Forestry and Environmental Resources, North Carolina State University, Raleigh, NC, 27607, United States; d Division of Infectious Diseases and Geographic Medicine, Stanford University, Stanford, CA, 94305, United States; e Environmental Intervention Unit, Infectious Disease Division, International Centre for Diarrhoeal Disease Research Bangladesh, Dhaka, 1212, Bangladesh; f Civil and Environmental Engineering, Tufts University, Medford, MA, 02153, United States

**Keywords:** 16S rRNA gene sequencing, SourceTracker, Low-and middle-income countries, Pathogen transmission, Fecal contamination

## Abstract

Understanding household-level transmission pathways of fecal pathogens can provide insight for developing effective strategies to reduce diarrheal illness in low- and middle-income countries. We applied whole bacterial community analysis to investigate pathways of bacterial transmission in 50 rural Bangladeshi households. SourceTracker was used to quantify the shared microbial community in household reservoirs (stored drinking water, soil, and hands) and estimate the percentage of fecal-associated bacteria from child and mothers’ feces in these reservoirs. Among the reservoirs studied, most bacterial transfer occurred between mothers’ and children’s hands and between mothers’ hands and stored water. The relative percentage of human fecal-associated bacteria in all household reservoirs was low. We also quantified the number of identical amplicon sequence variants within and between individual households to assess bacterial community exchange in the domestic environment. Intra-household sharing of bacteria between mothers’ and children’s hands and between hands and soil was significantly greater than inter-household sharing.

## Highlights

• 16S rRNA gene sequencing was used to examine bacterial communities in households.
• Low levels of fecal contamination were found on hands, in soil, and stored water.
• High proportion of bacteria was shared between mothers and their children.

## Graphical abstract

**Figure:**
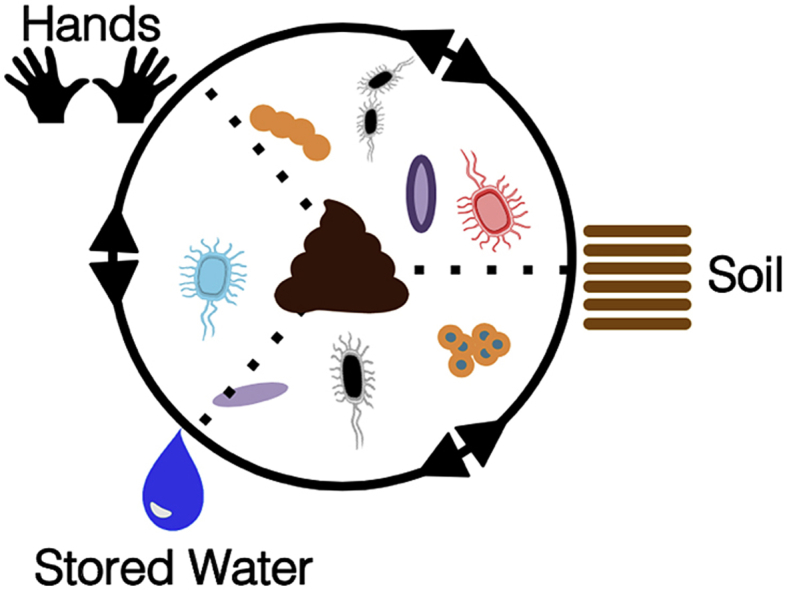

## Abbreviations

WASH: Water, sanitation, and hygiene
ASV: Amplicon sequence variant
LMIC: Low-and middle-income country
S: Soil
SW: Stored water
CH: Child hands
MH: Mother hands
CF: Child feces
MF: Mother feces

## 1 Introduction

Enteric pathogens are transmitted via the fecal-oral route through a variety of pathways in the environment. Pathogens can move through environmental reservoirs including flies, fomites, hands, soil, food, and water. Providing sufficient quantities of water, adequate drinking water treatment, sanitation, and hygiene (WASH) are the main strategies to block pathogen transmission pathways and reduce the occurrence of diarrheal illnesses. Recent field trials have investigated the impact of these interventions on diarrhea in children and found limited reductions (even with high levels of compliance) and no additive benefit of multiple interventions (Clasen et al., 2014; Humphrey et al., 2019; Luby et al., 2018, 2006; Null et al., 2018). Improving our understanding of the role of environmental reservoirs in enteric pathogen transmission is important, especially given the limited effectiveness of WASH interventions in the field.

Previous studies investigating environmental pathways of pathogen transmission have largely focused on identifying fecal indicators, such as *E. coli* and microbial source tracking markers, in household reservoirs such as soil, hands, stored drinking water, and children’s toys (Boehm et al., 2016; Ercumen et al., 2018b, 2018a; 2017; Harris et al., 2016; Mattioli et al., 2013; Odagiri et al., 2016; Pickering et al., 2010, 2012; 2011; Schriewer et al., 2015; Vujcic et al., 2014). This approach is useful because it can produce quantitative results for organisms that indicate the presence of fecal contamination. However, identifying traditional fecal indicators relies on pre-specification of single targets (bacteria) as an indicator of an entire microbial community (fecal microbial community). In microbial source tracking, which aims to distinguish fecal contamination from humans and various animals, the use of single host-specific targets has proven challenging because host microbiomes vary between individuals and geographically, impacting the sensitivity and specificity of assays when applied to populations outside of those used to develop these assays (Balleste et al., 2010; Gawler et al., 2007; Harris et al., 2016; Jenkins et al., 2009; Reischer et al., 2013).

Here, we aimed to characterize transmission pathways by applying untargeted high-throughput 16S rRNA gene sequencing to whole bacterial communities present in environmental samples. New and less expensive sequencing technologies have given rise to the study of a wide variety of microbiomes to understand bacterial transmission in the built environment. This research has revealed that microbiomes can be shared between environments, fomites, people, and animals (Pehrsson et al., 2016; Song et al., 2013; Trinh et al., 2018). Shared microbiota of concern includes pathogens, whereas shared non-pathogenic bacteria can have positive impacts on human health. For example, transmission of beneficial bacteria can be protective against pathogens and reduce the risk of developing allergies in young children (Jatzlauk et al., 2017; Peccia and Kwan, 2016; Prussin and Marr, 2015; Ursell et al., 2013).

To better understand shared microbiomes, SourceTracker (a Bayesian modeling tool for analyzing 16S rRNA gene amplicon sequence data), has been used to explore sources of fecal contamination in the natural environment as well as in the built environment by characterizing the shared microbiomes in homes, kitchens, hospitals, and restrooms (Flores et al., 2013, 2011; Lax et al., 2017; Ruiz-Calderon et al., 2016). SourceTracker uses characteristic sample types (provided by the user) to build a library of sequence profiles for each sample type. The sample type libraries can then be used to determine the attributable fraction of each sample type in unknown samples, distinct from those used to create the library. SourceTracker is particularly useful for sources in which there are no, or limited, single target markers that indicate the presence of the source (Knights et al., 2011). Despite the rapid onset of high throughput sequencing and subsequent computational tools such as SourceTracker, few studies have investigated household-level microbiomes in low-and middle-income countries (LMICs) (Bae et al., 2019; Bauza et al., 2019;Hospodsky et al., 2014 ; Mosites et al., 2017).

The goal of this study was to apply bacterial community analysis to elucidate pathways of enteric pathogen transmission in 50 rural Bangladeshi households. Specifically, we aimed to: 1) quantify the overlap in amplicon sequencing variants within and between individual households to distinguish intra-household bacterial community exchange; 2) quantify the shared microbial community in stored drinking water, soil, and child and mothers’ hands using SourceTracker; 3) estimate the percentage of human fecal-associated bacteria from child and mothers’ feces in household reservoirs, using SourceTracker, in an attempt to overcome the limitation of low host specificity exhibited by the single target markers in rural Bangladesh (Boehm et al., 2016); and 4) demonstrate the limits to identifying potential pathogens, at the genus and species level, using 16S rRNA gene sequencing in this context.

## 2 Methods

### 2.1 Environmental sample collection and processing

For this analysis, we had the opportunity to combine and sequence samples obtained from two studies conducted under the WASH Benefits randomized controlled trial in rural Bangladesh, which measured the impact of water, sanitation, hygiene, and nutrition interventions on child health outcomes (Luby et al., 2018). The environmental samples used herein are a subset of those in two previous manuscripts (Fuhrmeister et al., 2020, 2019) and were collected to assess the impact of improved sanitation on pathogens and indicators in the household environment. The fecal samples used herein are a subset of those in a forthcoming manuscript on the impact of WASH interventions on enteric infections (Grembi et al. n. d.).

50 control arm households, sampled as part of the environmental analysis, were selected for this study. Households were chosen based on availability of all environmental sample types (child hands (CH), mother hands (MH), stored water (SW), and soil (S)) collected at the same visit (Table 1). Detailed methods on environmental sample collection, DNA extraction, and qPCR are described elsewhere (Fuhrmeister et al., 2019). In brief, hand rinse samples were collected by participants placing their left-hand, or the left-hand of their child, into 250 mL of distilled water in a sterile Whirlpak bag (Nasco, Modesto, CA). Hands were massaged from the outside of the bag for 15 s, followed by 15 s of shaking. The same procedure was repeated with the right-hand in the same bag. Soil samples were collected by scraping the top layer of soil within a 30 × 30 cm^2^ stencil from an area as close to the house entrance as possible. Stored water samples were collected by asking mothers to provide a glass of water as they would give to their child under five. Samples were transported to the icddr,b field laboratory on ice and were processed within 12 h. At the field laboratory, 50 mL of hand rinse sample and up to 500 mL of stored water was filtered through a 0.45 μm HA filter (Millipore, Burlington, MA). Filters were treated with 0.5 mL of RNAlater and stored at −80 °C. Filter and soil samples were transported to UC Berkeley for DNA extraction and 16S rRNA gene sequencing.

**Table 1:** Sample types, sample size, and description of sample collection methods used in this study.

Sample Type	Sample No.	Description
**Household Samples**		
Child hands (CH)	50	Hand rinse obtained by massaging both hands in distilled water
Mother hands (MH)	49	Hand rinse obtained by massaging both hands in distilled water
Stored water (SW)	50	Water collected from household storage containers
Soil (S)	50	Top layer of soil scraped from an area near household entrance
**Additional Source Samples**		
Soil (S)	25	Soil negative for human and animal fecal markers
Child feces (CF)	25	Feces collected from children 13–19 months old
Mother feces (MF)	25	Feces collected from mothers during pregnancy

An additional 25 soil samples were selected from the control households for the source tracking soil library. To attempt to decouple the contributions from soil and feces, soil samples for the library were selected based on the absence of the human (HumM2) and animal (BacCow) fecal markers, as determined by qPCR. However, HumM2 had a relatively low sensitivity and specificity for human feces in rural Bangladesh, and it is possible human fecal contamination was missed by this marker (Boehm et al., 2016). Soil samples were imported to the U.S. in accordance with the USDA permit PPQ 525.

### 2.2 Fecal sample collection and processing

25 maternal fecal (MF) and 25 child fecal (CF) samples were obtained from households enrolled in the control and combined water, sanitation, and hygiene arms of WASH Benefits. Maternal and child fecal samples were not from the same households as those used for environmental analysis because fecal samples obtained for the enteric infection study (Grembi et al. n. d.) sampled different households than were sampled for the environmental analyses. Maternal fecal samples were collected during pregnancy. Mothers were instructed to collect their feces in sterile fecal collection containers and place the containers on ice in the evening before or morning of collection by field staff. Field staff transported fecal specimens on dry ice to the field laboratory. Child feces were collected on follow-up visits when children were between 13 and 19 months old using the same procedures. Specimens were stored at −80 °C until extraction. DNA was extracted from fecal samples using the QIAamp Fast DNA Stool Mini Kit (Qiagen, Germantown, MD), with an additional bead beating step. A blank sample was included in each round of extraction. Nucleic acid extract was transported on dry ice to Stanford University and then to UC Berkeley.

### 2.3 16S rRNA gene sequencing

Library preparation and sequencing was performed at the Vincent J. Coates Genomics Sequencing Laboratory at UC Berkeley. The V4 region of the 16S rRNA gene was amplified using 515f and 806r primers. Samples were pooled and sequenced on two MiSeq runs, yielding paired-end 250 bp reads. A mock community DNA standard (Zymo Research, Irvine, CA) was included on each MiSeq run. Approximately 13 million total reads were obtained from run one and 14 million reads from run two. An average of 50,460 reads per sample were obtained from child hands, 50,595 from mother hands, 87,160 from stored water, 89,514 from soil, 112,142 from child feces, and 117,675 from mother feces.

### 2.4 Data analysis

Forward and reverse reads were processed using the DADA2 pipeline (Callahan et al., 2016). Reads were truncated to 180 nucleotides, after which the quality score dropped significantly. The error rate was determined from sample reads and samples were denoised using the learned error model. Paired-end reads were merged to yield 250 bp sequences and chimeras were removed. On average 79% and 76% of the input reads from runs one and two remained after quality filtering, merging forward and reverse reads, and chimera removal. Taxonomy was assigned in DADA2 using a Naïve Bayes classifier that was trained on the Silva v132 database (Wang et al., 2007; Yilmaz et al., 2014). Species level identification was based on 100% identity between the reference database and amplicon sequence variants (ASVs) (Edgar, 2018b).

For pathogen specific analyses, we filtered ASVs at the genus and species level to bacterial pathogens associated with moderate to severe diarrhea in Bangladesh (*Escherichia coli, Aeromonas hydrophila*, *Campylobacter jejuni*, *Vibrio cholera*, and *Salmonella enterica*) (Kotloff et al., 2013). We included ASVs that matched multiple species if one of those was a pathogen. We omitted ASVs that were classified to the above genera but were assigned species that do not contain enteric pathogens.

Data were then analyzed using phyloseq (version 1.24.2) in R (version 3.5.0) (McMurdie and Holmes, 2013). A subset of samples was spiked with *Pseudomonas syringae* pv. *phaseolicola* (pph6) to estimate extraction efficiency in previous work (Fuhrmeister et al., 2019). This ASV was removed from all samples that were spiked. We also removed all ASVs associated with the eukaryotic organelles, chloroplasts and mitochondria (30,600 ASVs after organelle removal). In total, 6600 ASVs were found on child hands, 6300 ASVs on mother hands, 9300 ASVs in stored water, 12,300 ASVs in soil, 700 ASVs in child feces, and 1100 ASVs in mother feces. ASV abundance was normalized using the inverse hyperbolic sine transformation (Huber et al., 2002). Beta diversity was analyzed via PCoA using Bray-Curtis dissimilarity and variables contributing to differences between communities were identified with PERMANOVA using adonis in the vegan package for R (Oksanen et al., 2019). For all samples, we investigated the association between Bray-Curtis dissimilarity and sample type.

Overlapping ASVs (identical ASVs) within the same household, versus between different households, were determined by identifying the number of identical ASVs present in each sample type within the same household and in different households. To determine if the mean number of ASVs overlapping within and between households was significantly different, we used a bootstrap method (Efron and Tibshirani, 1993). The average number of ASVs matching between households in the real dataset was compared to the average number of ASVs matching in a randomly generated dataset (generated by randomizing household ID numbers) 10,000 times. For each iteration we calculated a test statistic which was the difference in the means between the number of ASVs matching in the same households in the real dataset and the number of ASVs matching between households in the randomly generated dataset. Statistical significance was determined depending on whether zero was included in the distribution of the test statistic at specified alpha values, 0.05 and 0.0083. The latter alpha value was adjusted to correct for multiple comparisons (CH and MH, CH and SW, CH and S, MH and SW, MH and S, and SW and S) using the Bonferroni correction (Abdi, 2007).

To estimate the association between bacterial communities in different reservoirs and feces, we used SourceTracker2 in python (version 3.7.0) (Knights et al., 2011). SourceTracker2 was run with default parameters and one-by-one, each environmental sample type was designated as a sink with all other environmental sample types and feces designated as sources. Fecal samples were used as sources for all models but in each run the reservoir of interest was removed from the source library and designated as a sink. For example, to investigate source associated ASVs on child hands, feces; mother hands; stored water; and soil were specified as sources and child hands was designated as the sink. We included all reservoirs in the source tracker analysis for consistency although some pairs do not represent realistic scenarios (e.g. the percentage of ASVs in soil sourced from hands). Student t-tests were used to compare the mean percentages assigned to different sources. To investigate whether SourceTracker results were driven by low abundance taxa, we conducted a sub-analysis in which SourceTracker was re-run after ASVs with an overall mean relative abundance less than 0.001% were removed (30,600 ASVs before and 5500 after filtering).

The source tracking analysis was validated using two approaches. In both, different percentages of DNA from samples of sources (child feces, mother feces, soil) and sinks (child hands, mother hands, stored water, soil) were prepared in the laboratory, and then compared to the percentages estimated using SourceTracker. All source and sink samples were composites, which were prepared by pooling equal masses of DNA extract from five individual samples of the same sample type. In validation approach one, DNA extract from each source (mother feces, child feces, and soil) was spiked into DNA extract for each sink (child hands, mother hands, or stored water) to achieve DNA concentration percentages of 10% source/90% sink, 1% source/99% sink and 0.5% source/99.5% sink. In approach two, child feces, mother feces, and soil source samples were combined in different DNA concentration percentages from 0 to 80%. All validation combinations are shown in Table S1. Samples used as sources were not included in SourceTracker source libraries.

## 3 Results

### 3.1 Quality controls

The relative abundance of the ZymoBIOMICS mock DNA community constituents was within 3 ± 2% between the two runs (Fig. S1). The eight most abundant taxa matched the reference sequences completely. Extraction blanks for environmental and fecal samples amplified poorly, or not at all, in PCR and were therefore not included in the pooled library. Note that the mother hand sample in household 48 was omitted due to poor PCR amplification.

### 3.2 Source tracking validation

There was good agreement between estimated source contributions and SourceTracker predicted contributions (Table S1). SourceTracker was sensitive to the lower percentages of source spike-in and was able to identify a qualitative difference between 1% and 0.5% (i.e. estimated contribution from 1% was consistently higher than the estimated contribution from 0.5% in the spike-in samples). When low abundant taxa (<0.001%) were removed from the SourceTracker analysis, the percentage of the spike-in samples that was attributed to soil (Table S2) was lower. In the source only samples, SourceTracker was able to correctly differentiate the relative contribution of different sources (mother feces, child feces, and soil). See supporting information for more details on validation results.

### 3.3 Beta diversity

**Fig. 1:**
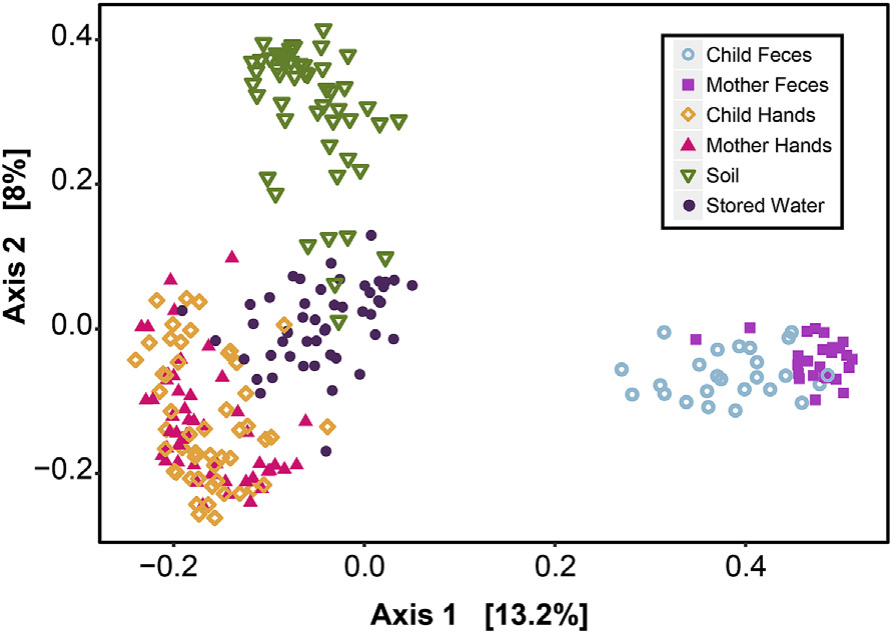
Principal coordinates analysis (PCoA) of bacterial community composition based on Bray-Curtis dissimilarity. Sample type is indicated by color and shape. (For interpretation of the references to color in this figure legend, the reader is referred to the Web version of this article.)

Sample type explained a significant difference in the bacterial community (Fig. 1) (PERMANOVA R^2^ = 0.27, p = 0.001). While samples appear to group based on sample type, it should be noted that there were some soil, stored water, and hand samples that cluster together and a low percentage of the variation was explained by the two axes (21%). In fecal samples, there was a significant difference in the communities between mothers and children (R^2^ = 0.22, p = 0.001). PERMANOVA tests were also performed using the Aitchison distance as suggested by Gloor et al. (2017). R^2^ values were similar and the difference in bacterial community by sample type was also statistically significant in these models.

### 3.4 Intra-household ASV matching

**Fig. 2:**
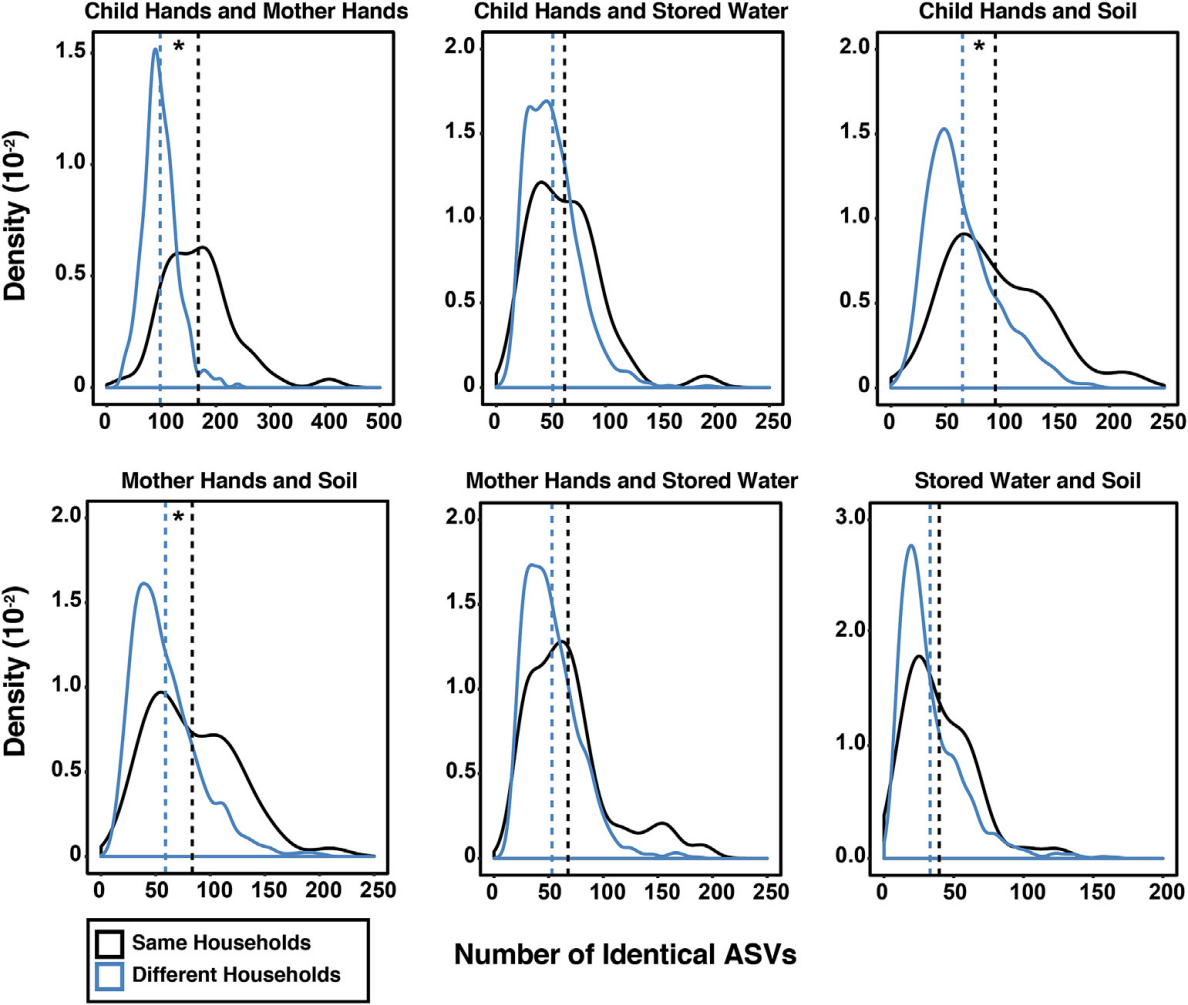
Density distribution of the number of identical ASVs in child hands and mother hands, child hands and stored water, child hands and soil, mother hands and soil, mother hands and stored water, and stored water and soil, for samples from the same households versus samples from different households. 50 households were sampled in total. Mean values for the number of ASVs matching within (same) and between (different) households are indicated with vertical dash lines. The asterisk indicates the difference within and between households was statistically significant, correcting for the multiple comparisons.

The average [95% CI] number of ASVs on mother hands, child hands, and in stored water and soil was 381 [337–425], 401 [352–451], 412 [362–462], and 698 [637–759] respectively. There were significantly more ASVs on child hands that were identical to mother hands within the same household (mean = 168), compared to the hands of mothers in other households (mean = 98) (Fig. 2). The lower bound of the distribution of the test statistic (difference in the means between the number of ASVs matching in the same households in the real dataset and the number of ASVs matching between households in the randomly generated dataset) was well above zero (95% CI: 51.7–80.2) (Fig. S2 and Table S3). The number of identical ASVs on mother and child hands and in soil was also significantly greater in the same household compared to other households. On average, 84 ASVs overlapped between mother hands and soil in the same household, while approximately 59 ASVs from mother hands matched soil from other households. Similarly, an average of 96 ASVs matched between child hands and soil within the same households, whereas an average of 65 ASVs from child hands matched with soil collected from other households. The lower bound of the test statistic distribution was above zero for both comparisons (95% CI CH to S: 14.9–41.3; MH to S: 10.2–35.3) (Table S3). There were no significant differences in the number of ASVs that were identical on mother hands and in stored water, on child hands and in stored water, and in soil and stored water within and between households, correcting for multiple comparisons.

### 3.5 SourceTracker

**Fig. 3:**
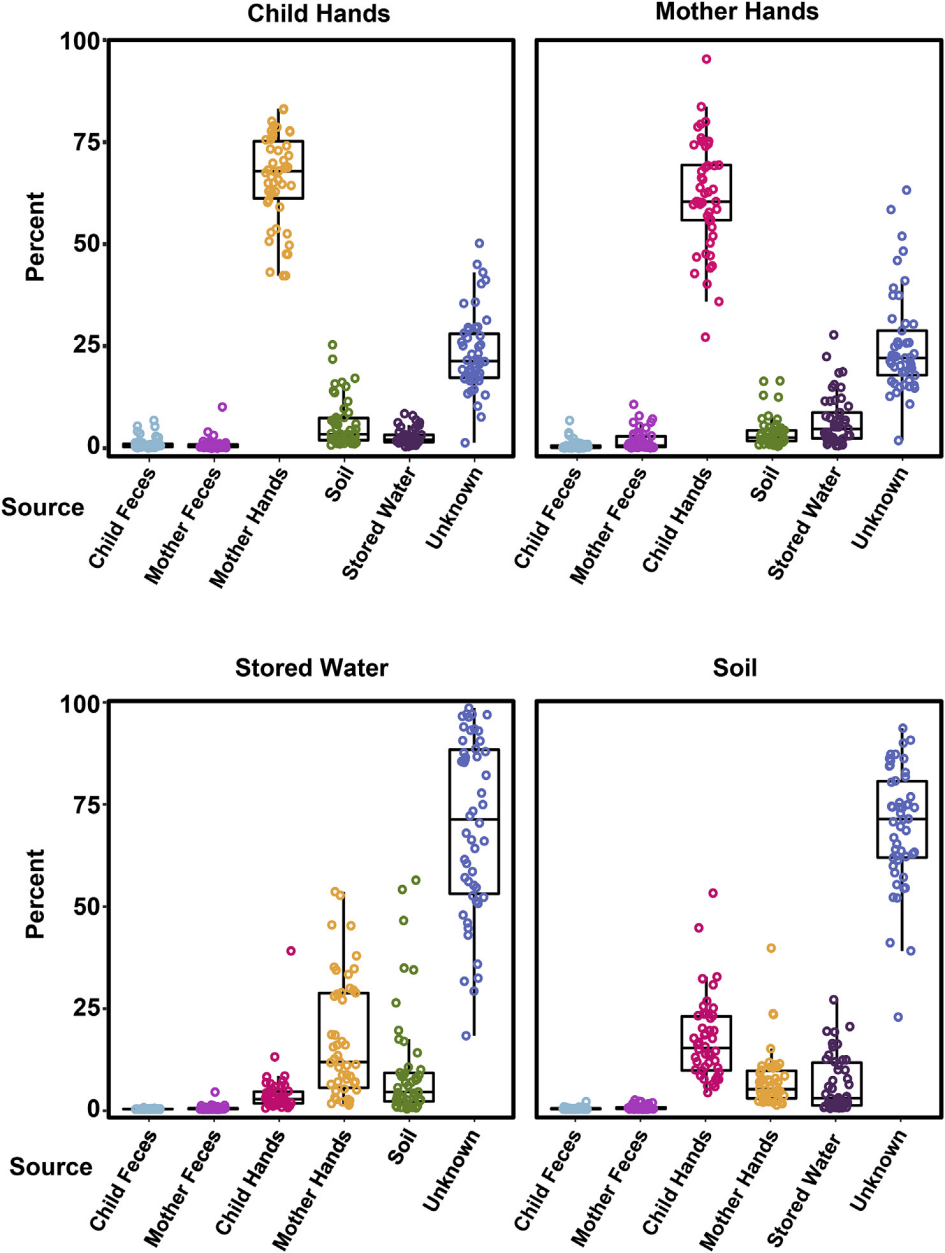
Percentage of bacteria in each sample that was associated with all other reservoir types, mothers’ feces and children’s feces as determined by SourceTracker. Each reservoir was included in the model for all other reservoirs for model consistency despite illogical pairs. Box plots indicate the overall distribution (25th percentile, median, 75th percentile, whiskers indicate at most 1.5x interquartile range).

Mother and child hands had the highest estimated percentage of associated bacteria of all reservoirs based on SourceTracker results (Fig. 3). On child hands over half of the bacteria, on average, were associated with mother hands (mean: 66.0 [95% CI: 63.0–69.1]%). Child feces and mother feces contributed approximately 1 percent (CF: 1.2 [0.8–1.6]%; MF: 0.9 [0.5–1.3]%). The estimated percentage from soil (5.8 [4.2–7.5]%) was significantly greater than child and mother feces (t-test, p < 0.001 for both). Similarly, on mother hands over 50% of the bacterial community was related to child hands, on average (61.8 [58.0–65.5]%). Although the percentage of fecal-associated ASVs was low (<2%), there was a statistically significant difference in the percentage associated with child feces and mother feces (CF: 0.7 [0.4–1.0]%; MF: 1.8 [1.2–2.5]%, p = 0.003). The percentage of ASVs on mother hands associated with soil (3.9 [2.8–4.9]%) was also significantly higher than that associated with child feces (p < 0.001).

In stored water, the estimated percentage of ASVs associated with mother and child feces was less than 0.5%. A greater percentage of the bacterial community was associated with hands (MH: 16.8 [12.8–20.8]%; CH: 3.8 [2.2–5.4]%), with a significantly greater percentage associated with mother hands (p < 0.001). On average 9.6 [5.9–13.3]% of the microbial community was related to soil, which was significantly greater than feces (p < 0.001 for both). However, the estimated percentage of ASVs associated with mother hands was significantly greater than the estimated percentage associated with soil (p = 0.01). In soil, less than 0.5% of the bacteria were human fecal-associated. When low abundant taxa were removed, the SourceTracker results were similar on mother and child hands (Fig. S3). In stored water, the percentage of ASVs attributed to unknown sources was higher (88.2%) and the percentage attributed to hands was lower (7.9%). It should be noted that the interpretation of associated bacteria between reservoirs is shared bacteria, however the term “shared” would be misleading here as association is determined by SourceTracker statistically based on probability, rather than direct occurrence.

### 3.6 Community constituents

*Sphingomonadaceae* was one of the most abundant families on hands, in soil, and in stored water (Figs. S4 and S5). Other abundant bacterial families on hands were common skin and environmental associated bacteria including *Moraxellaceae, Micrococcaceae, and Burkholderiaceae* (Fig. S4)*. Moraxellaceae and Burkholderiaceae* were also abundant families in stored water whereas *Chitinophagaceae, Rhodobacteraceae, and Xanthomonadaceae* were abundant in soil (Fig. S5).

We were unable to identify pathogens at the species level using 16S rRNA gene sequencing. Of the potentially pathogenic ASVs, 22 ASVs were identified from the genera *Campylobacter*, *Vibrio*, *Aeromonas*, *Salmonella*, and *Escherichia/Shigella* (Figs. S6 and S7). Of those, 16 were identified to the genus level only, four were identified to the species level in which pathogenic species were one of multiple possible species, and two were identified to a single genus and species (*E. coli* and *V. cholera*). The relative abundance of these potential pathogens was low in all sample types (<1%). We also assigned taxonomy using the RDP database (Cole et al., 2014) and taxonomic classifications of potential pathogen ASVs were the same at the genus level. The only differences were apparent in ASVs classified to multiple species; the assigned non-pathogenic species varied slightly between databases (data not shown).

## 4 Discussion

The goal of this study was to explore the usefulness of bacterial community analysis via 16S rRNA gene amplicon sequencing to provide insight into household-level transmission pathways of human feces and enteric pathogens. We explored the overlap of communities in different sample types within and between households to understand which intra-household transmission pathways were most likely to lead to shared ASVs. We then compared bacterial communities in different household sample types using SourceTracker. We also aimed to estimate the relative importance of human feces to the bacterial community in household reservoirs. Finally, we used the taxonomy assigned to ASVs to identify potential pathogens that were shared between different sample types within a household, at the genus and species level.

Using SourceTracker, we determined that the overall contribution of mother and child feces to the microbial communities was <2% on mother hands and child hands, and <0.5% in stored water and soil. In related previous work (Fuhrmeister et al., 2019), HumM2 was present on ≈20% of hands, in ≈20% of soil, and in <4% of stored water samples from 600 study households from the same study site. Although it is difficult to directly compare these results because presence of HumM2 does not have a quantitative value and SourceTracker attributed a very small fraction of bacteria in every sample to feces, there are some noteworthy comparisons. In Fig. 3, <0.5% of bacteria in stored water was sourced to human feces which is consistent with the very low abundance of HumM2 in the single marker study. In contrast, while <0.5% of bacteria in soil were sourced to feces, 20% of soil samples were positive for HumM2. Soil bacterial communities are more diverse than stored water, which could be the reason that despite the higher prevalence of HumM2 there was still only a small fraction of the bacteria in soil that were associated with human feces.

Our results are similar to those from a study of the bacterial community on hands of children, mothers and fathers in households in the United States (Shaffer et al., 2018). This previous study, which also used SourceTracker, found that the bacterial community on palms of children, mothers and fathers consisted of a median of 1.7% (range 0–99%) of fecal bacteria. They found that 12% of palms had over 25% fecal-associated bacteria, whereas none of the mother and child hands in our study had more than 10.7% of bacteria sourced to stool. It is notable that the percentage of fecal bacteria on hands in our study in a rural low-income area, with predominately pit latrines for sanitation, was not significantly greater than the percentage of fecal bacteria on hands in the high-income population. Although the relative proportion of fecal-associated bacteria is similar between studies, there is likely a difference in the pathogens present in feces. Even small amounts of fecal contamination are a health risk if they contain infectious pathogens. In previous work, hands of mothers and their children were contaminated with pathogenic *E. coli* and host-associated markers of fecal contamination in rural Bangladesh (Fuhrmeister et al., 2019).

The proportion of the microbial community on mother hands that was associated with mother feces was significantly greater than the percentage attributed to child feces (MF: 1.8% and CF: 0.7%, p = 0.003). This finding was unexpected given that disposal of child feces in Bangladesh typically involves caretaker handling of feces (Islam et al., 2018), and it contrasts a recent study which showed child feces was more dominant than adult feces on caregiver and child hands in Kibera, Kenya (Bauza et al., 2019). In our study, on child hands, which are important because children are more vulnerable to diarrheal illnesses, there was no significant difference between the percentage of the bacteria attributed to mother and child feces (CF: 1.2% and MF: 0.9%, p-value = 0.3). 3.9–5.8% of the bacteria on hands was soil-associated, and this contribution was greater than that from feces. Soil is a common flooring material in rural Bangladeshi households and children, especially of crawling age, make frequent hand contact with soil (Kwong et al., 2016).

The importance of the hand-soil pathway is also supported by the overlapping ASV analysis; more bacteria on mother and child hands were identical to bacteria in the soil in their own household compared to soil from other households. Soil has been shown to be contaminated with many different pathogens in LMICs (Baker et al., 2018; Pickering et al., 2012), including Bangladesh (Boehm et al., 2016; Fuhrmeister et al., 2019), and bacteria in soil can be readily transferred to the hands of children in the same household. Soil has also been shown to be a main environmental contributor to the bacterial community on caretaker hands in other LMICs, such as Tanzania (Hospodsky et al., 2014). In Tanzania, the most abundant families of bacteria on caretaker hands were soil-associated *Rhodobacteraceae* and *Nocardioidaceae*. The most abundant bacteria families in our study on mother hands were skin and environmental- associated *Moraxellaceae* followed by skin-associated *Micrococcaceae.*

The highest percentage of bacteria in the stored water microbial community was attributed to bacteria on mother hands (aside from the unknown category). In Bangladesh, water is typically collected from shallow tubewells and stored in a *kolshi* (metal container) that can be covered or uncovered. Water is then dispensed by pouring directly into a vessel for drinking or into an intermediary container, such as a pitcher, or by reaching into the container with a cup, which could result in the contamination of source water by mother hands. However, there was only a borderline significant difference in the number of identical bacteria on hands and in stored water within the same household compared to stored water from other households (Fig. 2 and Table S3). A related study among WASH Benefits households found that the concentration of indicator *E. coli* was higher in stored water compared to source water (Ercumen et al., 2018b), although growth of *E. coli* in stored water cannot be ruled out as the mechanism. Stored drinking water has also been studied using 16S rRNA gene amplicon sequencing in Cameroon, where the alpha diversity was higher in stored water compared to source water (Bae et al., 2019). The study theorized that the higher alpha diversity in stored water was due to introduction of bacteria from soil, humans, and air during storage. *Acintobacter*, a common water-related bacterium, was one of the most abundant genera in Cameroon and in our study (from the family *Moraxellaceae*).

Hand to hand transmission between mother and child is an important pathway for the exchange of bacteria. Looking at the overall microbial community composition, mother and child hands were very similar (Fig. 1, Fig. 3), which is unsurprising given that the two matrices can harbor similar skin-associated bacteria. However, there were significantly more identical bacteria on mother and child hands within the same households compared to other households, which suggests transfer of bacteria between mothers and children in the same household. Shared skin microbiomes have also been observed in families in the United States (Ross et al., 2017; Song et al., 2013), and we recommend including forehead swabs in future research to explicitly account for ASVs associated with the skin microbiome as a source (Shaffer et al., 2018). Although we identified very few ASVs that could be pathogens, we demonstrate the potential for pathogenic bacteria, if present, to be transferred through mother and child hand interactions. It should be noted that our analysis was unable to determine directionality or capture sharing via possible intermediaries, such as toys (fomites) between mothers and their children.

Of the potential pathogens we identified, almost half of the ASVs were from the genera *Campylobacter* which was not a pathogen investigated using targeted analysis (qPCR and PCR) in related work (Fuhrmeister et al., 2019), but has been detected in children in Bangladesh (Grembi et al. n. d.; Kotloff et al., 2013; Platts-Mills et al., 2014). In resource constrained areas, where the burden of diarrhea illness is high and there are numerous etiological agents of disease, 16S rRNA gene could be a useful screening tool prior to selection of PCR targets. For example, in retrospect pathogenic *Campylobacter* spp. would have been an interesting target, and we could have made this choice if we had conducted the 16S rRNA gene sequencing in advance of selecting qPCR targets. However, it should be emphasized that it was not possible to identify actual pathogens using 16S rRNA gene sequencing. For most species, only a fraction of strains or subtypes are pathogenic to humans. In our analysis, the only two ASVs identified to the species level that could be potential pathogens were *E. coli* and *V. cholera*, both of which have numerous non-pathogenic subtypes. In addition, while DADA2 assigns species at 100% identity, the V4 region of the 16S rRNA gene is not enough to differentiate many strains. More genomic context is needed to identify pathogenic strains, which is possible with whole genome sequencing (metagenomics) or qPCR. However, in order to identify pathogens that are present in low relative abundance in environmental samples, considerably higher sequencing depth is required using metagenomics compared to amplicon methods, significantly increasing cost. Additionally, assigning species relies on the accuracy of existing databases. We found no difference in the taxonomy assigned to potential pathogen ASVs at the genus level between Silva and RDP, but a previous study estimated annotation error rates in these databases to be 17% and 10%, respectively (Edgar, 2018a). Another limitation is that only viable organisms are capable of causing illness and viability cannot be determined from 16S rRNA gene sequencing alone. Therefore, results from 16S rRNA gene sequencing analysis should be coupled with specific and sensitive pathogen detection methods (e.g., qPCR), and culture-based methods to determine viability, when feasible.

While we observed quantitative differences in the contribution of varying sources, further validation of this approach is needed, especially for sources with estimated low percent contributions. Unlike previous work that used SourceTracker to identify sources of contamination based on threshold values, we have included all SourceTracker results in our analysis and have avoided using thresholds for assigning a source. In Bauza et al. a 1% threshold was used as the cut off value to consider samples positive for source fecal contamination or if the source was positive in at least 3 of 5 runs with an average relative standard deviation less than 100% (Bauza et al., 2019). Although there is still uncertainty about how sensitive SourceTracker is to small quantities of fecal contamination in varying contexts, previous work found that SourceTracker correctly identified fecal sources with as little as 0.025% (vol/vol) of feces in river water in the United States (Staley et al., 2018). However, SourceTracker percentages were consistently higher than what was expected based on vol/vol ratios (Staley et al., 2018). In our own work, validation samples were composed of DNA mass ratios (post DNA extraction), making it difficult to compare strategies for determining sensitivity as the amount of DNA that can be extracted from specific quantities of feces is unknown, and highly variable. Another inconsistency between validation studies is the composition of the unknown samples. Complexity of bacterial communities varies by sample type (river water, hand rinse, soil) and is likely to impact SourceTracker performance. Evaluating the sensitivity of SourceTracker in different sample types is important given that low levels of fecal contamination are relevant for public health (due to the low infectious doses of pathogens).

A limitation of this study design is that it relied on repurposed samples originally collected for other related work (Fuhrmeister et al., 2020, 2019) and a WASH Benefits stool study (Grembi et al. n. d.). As a result, we were constrained to the environmental sample types collected for the previous work and fecal samples that were not from the same households as the environmental samples. In future work, we recommend collecting more bacterial reservoirs in the household to include in the analysis. For example, a high percentage of bacteria in stored water was sourced from unknown sample types (did not come from human feces or other environmental sample types). A portion of this unknown bacterial community could have been from animal fecal contamination, which was not included in our study. Animal feces from cows, goats, ducks, and chickens is common in rural Bangladeshi households and has been associated with increased concentrations of fecal indicator organisms as well as increased prevalence of enteric pathogens (Ercumen et al., 2017; Fuhrmeister et al., 2019). Sampling other microbiomes such as the forehead and mouth could improve the ability to account for more of the bacteria found on hands (Shaffer et al., 2018), and sampling groundwater could improve the accounting for stored water. Also, in our existing analysis of intra-household transmission, identifying the number of identical ASVs does not take into account relative abundance, which is included in SourceTracker models. Therefore, minor community members are represented equally with major constituents and the number of ASVs could be influenced by sequencing depth which resulted in more ASVs in soil than stored water and hands. Using SourceTracker to investigate transmission pathways is also complicated by the inability to determine directionality. For example, mother hands and child hands had the highest number of associated ASVs but it is unknown what proportion of the bacteria originated from mothers versus children. Lastly, SourceTracker is less able to distinguish sources that have more similar microbial communities (Bauza et al., 2019; Staley et al., 2018). While the sample types in this study had distinct microbial communities, including child and mother feces, there was still some overlap and this study only captured some of the factors that may influence the microbial communities. This limitation is evident in the low percentage of the variation (total of 21%) that was explained by Axis 1 and 2 in the multidimensional scaling (Fig. 1).

Bacterial community analysis is a promising approach for understanding pathways of enteric pathogen transmission. Although we show relatively low proportions of human fecal bacteria in the bacterial community in environmental reservoirs, low levels of fecal contamination can still transmit pathogens. Nonetheless, we were able to identify pathways of bacteria transfer between mother and child hands, soil and child hands, and soil and mother hands at the household level. This non-targeted approach may be more appropriate to use in LMIC settings where sensitivity and specificity of source tracking markers is impacted by the potential sharing of microbial communities between humans and animals. Even though sharing of microbial communities between humans and animals is also a limitation in differentiating sources using SourceTracker, the use of multiple markers as indicators of fecal contamination could be more robust to similarity in source microbial communities than single targets, and further exploration is warranted.

## 5 Conclusions

• Only a small fraction of the bacterial community in household reservoirs was human fecal-associated, although low levels of fecal contamination can still pose a health risk.
• The percentage of fecal-associated ASVs on hands in rural Bangladesh was similar to the percentage of fecal-associated bacteria found on hands in the US in a previously published study.
• Mother and child hands had the highest percentage of associated bacteria and significantly more bacteria were identical between mother and child hands within the same household, compared to those from other households.
• In stored water, the highest percentage of bacteria from a known source was associated with mother hands.
• The percentage of ASVs from unknown sources was high and future work should include more household source types.

## Data Availability

Raw sequence reads for this study were deposited into the sequence read archives under BioProject PRJNA604825.

## Declaration of competing interest

The authors declare that they have no known competing financial interests or personal relationships that could have appeared to influence the work reported in this paper.
