# Supplementary material for: Shared bacterial communities between soil, stored drinking water, and hands in rural Bangladeshi households
Source: Water Res X. 2020 May 23;9:100056. doi: 10.1016/j.wroa.2020.100056 (PMC7276488; doi:10.1016/j.wroa.2020.100056)
Supplement: Multimedia component 1 [file mmc1.docx]

**Supporting Information**

**Shared Bacterial Communities Between Soil, Stored Drinking Water, and Hands in Rural Bangladeshi Households**

Erica R. Fuhrmeister^a^, Ayse Ercumen^b,c^, Jessica Grembi^d^, Mahfuza Islam^e^, Amy J. Pickering^f^, Kara L. Nelson^a*^

^a^Department of Civil and Environmental Engineering, University of California, Berkeley, California, 94720, United States

^b^School of Public Health, University of California, Berkeley, California, 94720, United States

^c^Department of Forestry and Environmental Resources, North Carolina State University, Raleigh, North Carolina, 27607, United States

^d^Division of Infectious Diseases and Geographic Medicine, Stanford University, Stanford, California, 94305, United States

^e^Infectious Disease Division, International Centre for Diarrhoeal Disease Research Bangladesh, Dhaka, 1212, Bangladesh

^f^Civil and Environmental Engineering, Tufts University, Medford, Massachusetts, 02153, United States

*Correspondence to:

karanelson@berkeley.edu

CEE Dept MS 1710

University of California

Berkeley, California 94720

Figures: 7

Tables: 3

Pages: 11

**SourceTracker Validation**

The validation analysis was conducted with (Table S1) and without low abundance taxa (<0.001)% (Table S2). In both analyses there was a strong linear relationship between the estimated and predicted source percentages (All ASVs: Pearson's rho = 0.95, 0.95, 0.77 for child feces, mother feces and soil p< 0.001 for all; low abundance filtered: Pearson's rho = 0.97, 0.95, 0.92 for child feces, mother feces and soil p< 0.001 for all). One challenge in preparing the spike-in samples is that we were unable to identify multiple hand rinse and stored water samples to use as a sink that tested negative for HumM2 and BacCow markers; soil samples used as a sink were negative for both fecal markers. Therefore it should be noted that our calculations for the percentage of sources in the validation samples only account for the amount of DNA added to the samples; the presence of child feces, mother feces, and soil DNA in the validation samples of mother hand, child hand and stored water samples could have caused the actual contributions to differ from the prepared percentages. This possibility is real given that HumM2 and BacCow markers were present in some of the environmental samples that were used to prepare the sink samples. This resulted in what appears to be false positives, but in fact the actual percentage of child or mother fecal DNA in Table S1 is likely non-zero for mixes that contained hand rinse or stored water DNA. When low abundant taxa are filtered, the percentage of soil-associated ASVs in the spike-in samples is lower than when low abundant taxa are not removed. This difference is likely due to the high diversity in the microbial community of soil.

In the source only samples, SourceTracker predicted low percentages from soil and mother feces when soil and mother feces were not part of the source samples (0-5.0%) (Table S1). In samples without child feces, SourceTracker predicted slightly greater contributions (6.4-9.3%) from child feces. Soil was also consistently underestimated in the observed contribution compared to the expected.

| **Table S1**: Source Tracker was validated using two different approaches. Each row reports a sample prepared in the laboratory with unique percent contribution of sources and sinks, which is compared to percent contribution (standard deviation) estimated by SourceTracker. 1) DNA extract from sources (mother feces, child feces, and soil) was spiked into child hands, mother hands, and stored water. Source samples were spiked into the environmental sink samples to achieve DNA concentration ratios of 10% source/90% sink, 1% source/99% sink, and 0.5% source/99.5% sink. 2) Child feces, mother feces, and soil source samples were combined in different DNA concentration ratios from 0 to 80% (no sink). | | | | | | | | | | | |
| --- | --- | --- | --- | --- | --- | --- | --- | --- | --- | --- | --- |
| **Laboratory Preparation** | | | | | | | | **Source Tracker Estimates % (Std)** | | | |
| Source | % | Source | % | Source | % | Sink | % | CF | MF | Soil | Unknown |
| 1. Spike-In | | | | | | | | | | | |
| CF | 0 | MF | 10.0 | Soil | 0 | SW C | 90.0 | 2.4 (0.5) | 18.1 (0.5) | 14.1 (0.2) | 65.4 (0.4) |
| CF | 0 | MF | 1.0 | Soil | 0 | SW C | 99.0 | 0.8 (0.2) | 2.7 (0.2) | 19.7 (0.2) | 76.9 (0.2) |
| CF | 0 | MF | 0.5 | Soil | 0 | SW C | 99.5 | 0.3 (0.1) | 1.1 (0.1) | 20.8 (0.2) | 77.8 (0.2) |
| CF | 10.0 | MF | 0 | Soil | 0 | SW C | 90.0 | 12.9 (0.5) | 0.6 (0.2) | 17.3 (0.3) | 69.2 (0.5) |
| CF | 1.0 | MF | 0 | Soil | 0 | SW C | 99.0 | 2.8 (0.1) | 0.1 (0.1) | 24.1 (0.2) | 73.0 (0.3) |
| CF | 0.5 | MF | 0 | Soil | 0 | SW C | 99.5 | 1.3 (0.1) | 0.1 (0.1) | 22.4 (0.2) | 76.2 (0.2) |
| CF | 10.0 | MF | 0 |  |  | S 357 | 90.0 | 16.2 (0.4) | 1.2 (0.3) | 56.2 (0.3) | 26.3 (0.5) |
| CF | 1.0 | MF | 0 |  |  | S 357 | 99.0 | 2.5 (0.2) | 0.7 (0.3) | 63.5 (0.5) | 33.4 (0.6) |
| CF | 0.5 | MF | 0 |  |  | S 357 | 99.5 | 2.0 (0.2) | 0.7 (0.1) | 66.8 (0.4) | 30.6 (0.5) |
| CF | 10.0 | MF | 0 | Soil | 0 | CH C | 90.0 | 18.9 (0.3) | 2.3 (0.2) | 40.7 (0.8) | 38.1 (0.8) |
| CF | 1.0 | MF | 0 | Soil | 0 | CH C | 99.0 | 4.7 (0.2) | 1.7 (0.3) | 20.7 (1.9) | 73.0 (1.8) |
| CF | 0.5 | MF | 0 | Soil | 0 | CH C | 99.5 | 2.7 (0.2) | 0.8 (0.2) | 21.8 (1.5) | 74.7 (1.6) |
| CF | 10.0 | MF | 0 | Soil | 0 | MH C | 90.0 | 22.6 (0.6) | 2.2 (0.4) | 13.9 (0.3) | 61.3 (0.5) |
| CF | 1.0 | MF | 0 | Soil | 0 | MH C | 99.0 | 21.2 (0.4) | 3.3 (0.4) | 17.3 (0.5) | 58.3 (0.9) |
| CF | 0.5 | MF | 0 | Soil | 0 | MH C | 99.5 | 1.7 (0.3) | 1.4 (0.3) | 22.8 (0.4) | 74.1 (0.4) |
| CF | 0 | MF | 0 | Soil | 10.0 | SW C | 90.0 | 0.1 (0.1) | 0.2 (0.1) | 25.7 (0.2) | 74.0 (0.2) |
| CF | 0 | MF | 0 | Soil | 1.0 | SW C | 99.0 | 0.3 (0.1) | 0.2 (0.1) | 20.7 (0.3) | 78.8 (0.3) |
| CF | 0 | MF | 0 | Soil | 0.5 | SW C | 99.5 | 0.1 (0.1) | 0.2 (0.0) | 20.4 (0.2) | 79.4 (0.2) |
| CF | 10.0 | MF | 10.0 | Soil | 10.0 | SW C | 70.0 | 17.2 (0.5) | 16.2 (0.5) | 15.3 (0.2) | 51.4 (0.3) |
| CF | 1.0 | MF | 1.0 | Soil | 1.0 | SW C | 97.0 | 2.7 (0.2) | 1.8 (0.3) | 20.8 (0.3) | 74.7 (0.3) |
| 1. Source Composites Only | | | | | | | | | | | |
| CF | 10.0 | MF | 80.0 | Soil | 10.0 |  |  | 19.2 (0.9) | 51.6 (0.9) | 2.8 (0.1) | 26.4 (0.4) |
| CF | 80.0 | MF | 10.0 | Soil | 10.0 |  |  | 63.0 (0.7) | 20.2 (0.6) | 4.8 (0.2) | 12.0 (0.3) |
| CF | 10.0 | MF | 10.0 | Soil | 80.0 |  |  | 17.6 (0.8) | 21.6 (0.8) | 39.2 (0.3) | 21.6 (0.5) |
| CF | 0.0 | MF | 50.0 | Soil | 50.0 |  |  | 8.0 (0.8) | 49.7 (0.9) | 14.8 (0.2) | 27.5 (0.5) |
| CF | 50.0 | MF | 0.0 | Soil | 50.0 |  |  | 50.2 (0.5) | 3.2 (0.6) | 31.2 (0.3) | 15.4 (0.5) |
| CF | 50.0 | MF | 50.0 | Soil | 0.0 |  |  | 37.3 (0.9) | 41.2 (0.8) | 0.0 (0.0) | 21.6 (0.2) |
| CF | 0.0 | MF | 25.0 | Soil | 75.0 |  |  | 6.4 (0.7) | 36.2 (0.7) | 30.7 (0.1) | 26.7 (0.2) |
| CF | 0.0 | MF | 75.0 | Soil | 25.0 |  |  | 9.3 (0.8) | 55.2 (0.9) | 8.2 (0.1) | 27.3 (0.4) |
| CF | 25.0 | MF | 0.0 | Soil | 75.0 |  |  | 35.9 (0.5) | 1.5 (0.5) | 43.6 (0.4) | 19.0 (0.3) |
| CF | 75.0 | MF | 0.0 | Soil | 25.0 |  |  | 68.8 (0.4) | 5.0 (0.4) | 17.1 (0.2) | 9.0 (0.5) |
| CF | 75.0 | MF | 25.0 | Soil | 0.0 |  |  | 51.4 (0.6) | 33.2 (0.8) | 0.0 (0.0) | 15.4 (0.3) |
| CF | 25.0 | MF | 75.0 | Soil | 0.0 |  |  | 22.7 (1.0) | 51.3 (1.0) | 0.0 (0.0) | 26.1 (0.3) |

| **Table S2**: Source Tracker was validated using two different approaches. Each row reports a sample prepared in the laboratory with unique percent contribution of sources and sinks, which is compared to percent contribution (standard deviation) estimated by SourceTracker. 1) DNA extract from sources (mother feces, child feces, and soil) was spiked into child hands, mother hands, and stored water. Source samples were spiked into the environmental sink samples to achieve DNA concentration ratios of 10% source/90% sink, 1% source/99% sink, and 0.5% source/99.5% sink. 2) Child feces, mother feces, and soil source samples were combined in different DNA concentration ratios from 0 to 80% (no sink). Low abundance taxa (<0.001%) were filtered out. | | | | | | | | | | | |
| --- | --- | --- | --- | --- | --- | --- | --- | --- | --- | --- | --- |
| **Laboratory Preparation** | | | | | | | | **Source Tracker Estimates % (Std)** | | | |
| Source | % | Source | % | Source | % | Sink | % | CF | MF | Soil | Unknown |
| 1. Spike-In | | | | | | | | | | | |
| CF | 0 | MF | 10.0 | Soil | 0 | SW C | 90.0 | 2.2 (0.2) | 14.3 (1.2) | 1.0 (0.4) | 82.5 (1.3) |
| CF | 0 | MF | 1.0 | Soil | 0 | SW C | 99.0 | 0.4 (0.2) | 2.3 (0.3) | 1.2 (0.3) | 96.1 (0.4) |
| CF | 0 | MF | 0.5 | Soil | 0 | SW C | 99.5 | 0.4 (0.2) | 0.4 (0.2) | 1.0 (0.3) | 98.3 (0.4) |
| CF | 10.0 | MF | 0 | Soil | 0 | SW C | 90.0 | 11.0 (0.2) | 0.8 (0.3) | 1.6 (0.4) | 86.7 (0.5) |
| CF | 1.0 | MF | 0 | Soil | 0 | SW C | 99.0 | 1.2 (0.1) | 0.1 (0.1) | 1.4 (0.3) | 97.4 (0.3) |
| CF | 0.5 | MF | 0 | Soil | 0 | SW C | 99.5 | 0.5 (0.1) | 0.0 (0.0) | 1.1 (0.4) | 98.4 (0.4) |
| CF | 10.0 | MF | 0 |  |  | S 357 | 90.0 | 16.3 (0.5) | 0.8 (0.3) | 52.8 (0.4) | 30.2 (0.6) |
| CF | 1.0 | MF | 0 |  |  | S 357 | 99.0 | 2.8 (0.2) | 0.4 (0.1) | 63.3 (0.5) | 33.5 (0.5) |
| CF | 0.5 | MF | 0 |  |  | S 357 | 99.5 | 1.7 (0.1) | 0.6 (0.2) | 62.0 (0.7) | 35.7 (0.7) |
| CF | 10.0 | MF | 0 | Soil | 0 | CH C | 90.0 | 17.3 (0.3) | 0.5 (0.2) | 3.5 (0.5) | 78.8 (0.6) |
| CF | 1.0 | MF | 0 | Soil | 0 | CH C | 99.0 | 2.7 (0.4) | 0.2 (0.2) | 4.0 (1.1) | 93.1 (1.3) |
| CF | 0.5 | MF | 0 | Soil | 0 | CH C | 99.5 | 2.0 (0.2) | 0.3 (0.2) | 3.1 (0.8) | 94.6 (0.9) |
| CF | 10.0 | MF | 0 | Soil | 0 | MH C | 90.0 | 19.4 (0.5) | 1.1 (0.3) | 12.8 (1.1) | 66.8 (1.3) |
| CF | 1.0 | MF | 0 | Soil | 0 | MH C | 99.0 | 18.1 (0.4) | 0.9 (0.2) | 12.3 (1.2) | 68.8 (1.3) |
| CF | 0.5 | MF | 0 | Soil | 0 | MH C | 99.5 | 0.8 (0.2) | 1.4 (0.3) | 11.0 (0.9) | 86.8 (1.0) |
| CF | 0 | MF | 0 | Soil | 10.0 | SW C | 90.0 | 0.0 (0.0) | 0.3 (0.1) | 4.9 (1.0) | 94.9 (1.1) |
| CF | 0 | MF | 0 | Soil | 1.0 | SW C | 99.0 | 0.0 (0.0) | 0.2 (0.1) | 1.8 (0.2) | 98.0 (0.3) |
| CF | 0 | MF | 0 | Soil | 0.5 | SW C | 99.5 | 0.1 (0.1) | 0.1 (0.1) | 1.3 (0.5) | 98.5 (0.5) |
| CF | 10.0 | MF | 10.0 | Soil | 10.0 | SW C | 70.0 | 14.1 (0.5) | 15.3 (0.7) | 4.0 (1.0) | 66.6 (1.0) |
| CF | 1.0 | MF | 1.0 | Soil | 1.0 | SW C | 97.0 | 2.4 (0.3) | 2.5 (0.3) | 1.6 (0.5) | 93.6 (0.6) |
| 1. Source Composites Only | | | | | | | | | | | |
| CF | 10.0 | MF | 80.0 | Soil | 10.0 |  |  | 20.8 (1.0) | 55.9 (0.9) | 1.8 (0.2) | 21.5 (1.0) |
| CF | 80.0 | MF | 10.0 | Soil | 10.0 |  |  | 63.0 (0.9) | 23.1 (1.0) | 5.7 (0.1) | 8.2 (0.4) |
| CF | 10.0 | MF | 10.0 | Soil | 80.0 |  |  | 17.7 (1.0) | 20.4 (0.6) | 34.5 (0.6) | 27.4 (1.0) |
| CF | 0.0 | MF | 50.0 | Soil | 50.0 |  |  | 6.8 (1.1) | 48.5 (1.0) | 15.6 (0.6) | 29.0 (1.1) |
| CF | 50.0 | MF | 0.0 | Soil | 50.0 |  |  | 44.2 (0.6) | 2.5 (0.6) | 28.3 (0.4) | 25.1 (0.8) |
| CF | 50.0 | MF | 50.0 | Soil | 0.0 |  |  | 42.0 (1.3) | 44.5 (1.1) | 0.0 (0.0) | 13.5 (0.4) |
| CF | 0.0 | MF | 25.0 | Soil | 75.0 |  |  | 6.2 (1.4) | 38.2 (1.5) | 29.6 (0.5) | 26.1 (0.8) |
| CF | 0.0 | MF | 75.0 | Soil | 25.0 |  |  | 7.5 (1.2) | 56.4 (1.1) | 6.1 (0.3) | 30 (0.7) |
| CF | 25.0 | MF | 0.0 | Soil | 75.0 |  |  | 31.8 (0.5) | 1.3 (0.4) | 40.4 (0.5) | 26.6 (0.7) |
| CF | 75.0 | MF | 0.0 | Soil | 25.0 |  |  | 63.2 (0.9) | 2.5 (0.6) | 14.6 (0.3) | 19.8 (0.8) |
| CF | 75.0 | MF | 25.0 | Soil | 0.0 |  |  | 55.2 (0.9) | 34.1 (0.9) | 0.0 (0.0) | 10.7 (0.3) |
| CF | 25.0 | MF | 75.0 | Soil | 0.0 |  |  | 29.7 (0.7) | 50.3 (1.1) | 0.0 (0.0) | 20 (1.3) |

**Figure S1**: Relative abundance of mock community members for each sequencing run in comparison to the theoretical relative abundances from ZymoBIOMICS. The eight most abundant taxa matched the reference sequences completely and the next most abundant ASV in both lanes (1.4% of sample reads in both mock communities) had a one bp mismatch from the reference sequence of *Salmonella enterica.*


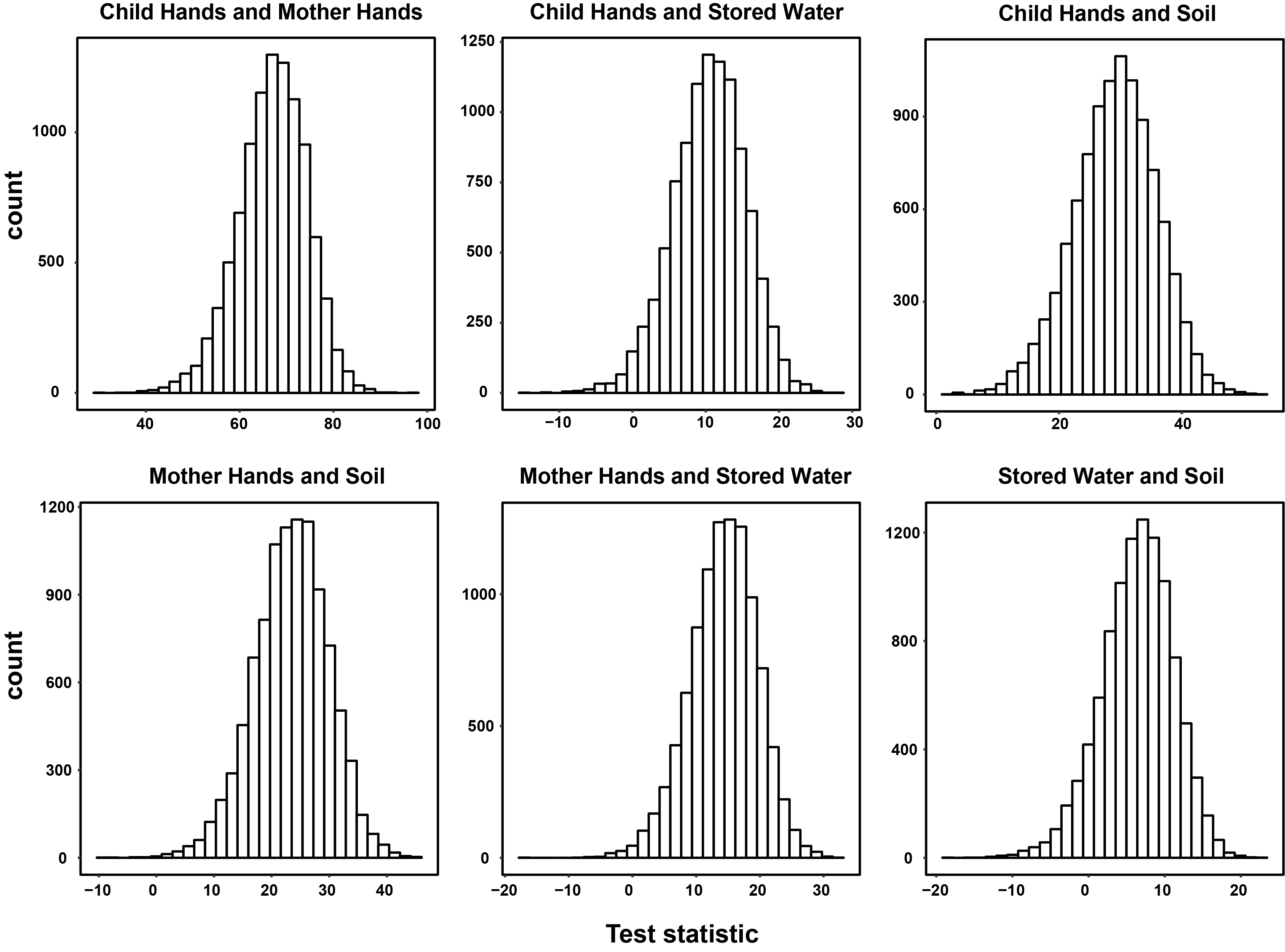


**Figure S2**: Distribution of the test-statistic (difference in mean between the number of intra-household ASVs in bootstrapped iterations of randomly assigned households and actual data). See Table S3 for confidence intervals.

| **Table S3**: 95% CI and 99.15% confidence interval of the test-statistic (difference in mean between the number of identical intra-household ASVs in bootstrapped iterations of randomly assigned households and actual data). Distributions that do not include zero are statistically significant at alpha=0.05 and 0.0083. Alpha=0.0083 is corrected for multiple comparisons. | | |
| --- | --- | --- |
|  | T-statistic distribution  2.5-97.5% CI [alpha =0.05] | T-statistic distribution  0.42-99.58%CI [alpha=0.0083] |
| Child hands to mother hands | 51.7 - 80.2 | 45.1 – 84.6 |
| Child hands to stored water | 0.3 - 19.5 | -4.4 – 22.7 |
| Child hands to soil | 14.9 - 41.3 | 10.0 – 45.7 |
| Mother hands to stored water | 3.1 - 24.0 | -1.1 - 27.3 |
| Mother hands to soil | 10.2 - 35.3 | 4.2 - 39.8 |
| Stored water to soil | -3.6 - 15.0 | -8.2 - 17.4 |

**Figure S3**: Percentage of bacteria in each sample that was associated with all other reservoir types, mothers’ feces, and child feces as determined by SourceTracker. Each reservoir was included in the model for all other reservoirs for model consistency despite illogical pairs. Box plots indicate the overall distribution (25th percentile, median, 75th percentile. Whiskers indicate at most 1.5x interquartile range). Low abundance taxa (<0.001%) were filtered out.


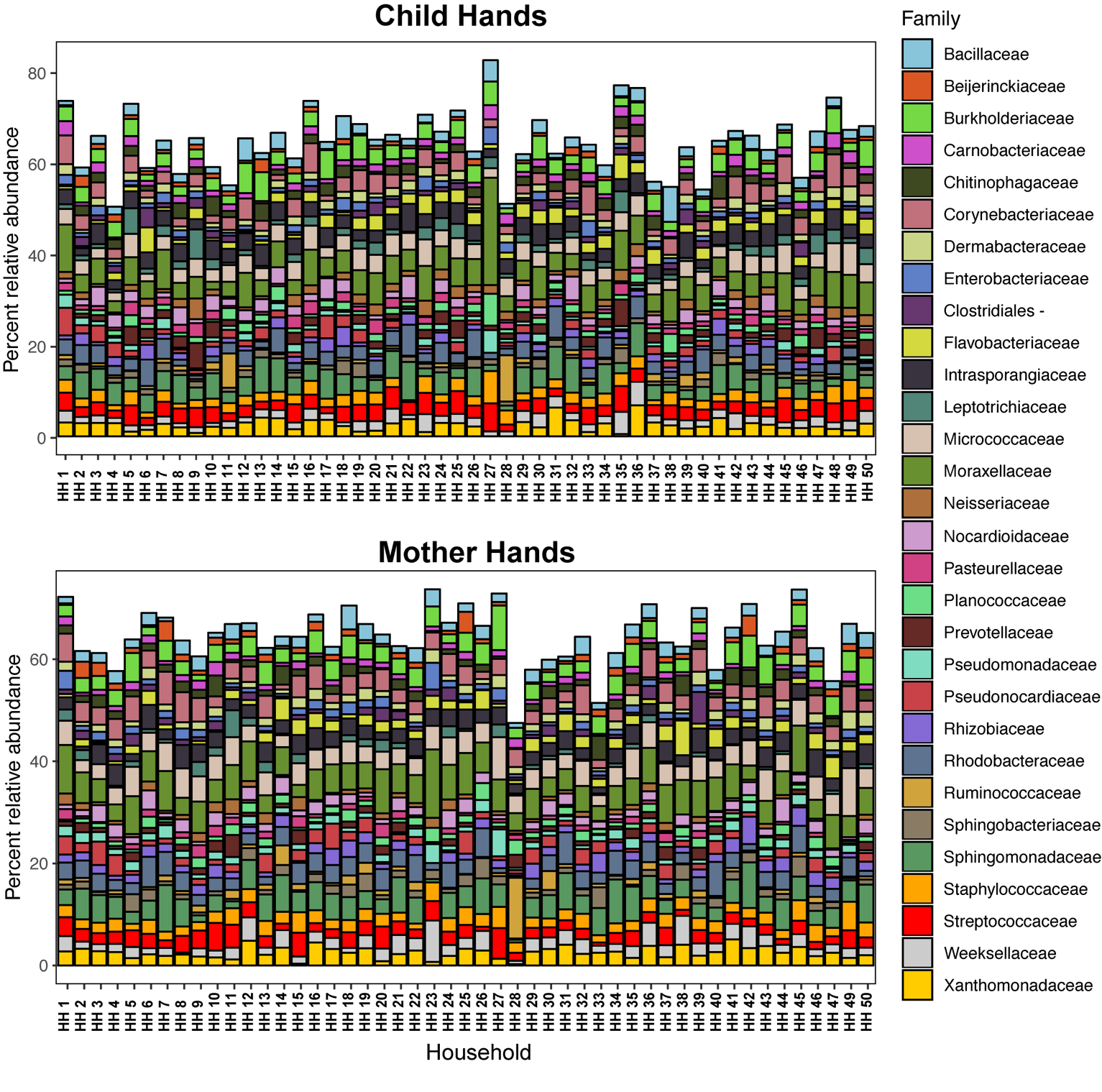


**Figure S4**: Relative abundance (percentage of total sample reads) of the 30 most abundant families on hands.


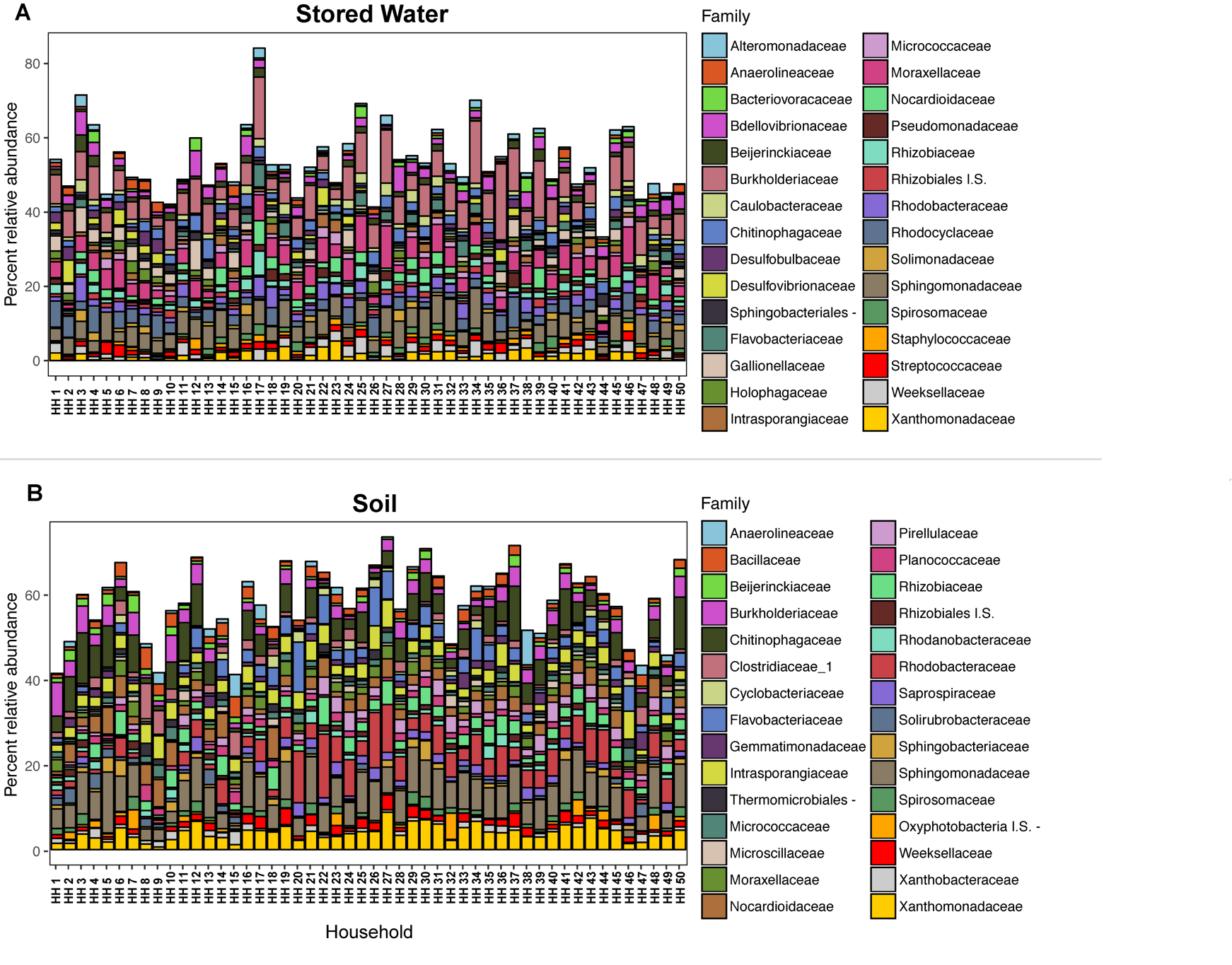
**Figure S5**: A.) Relative abundance (percentage of total sample reads) of the 30 most abundant families in stored water. B.) Relative abundance (percentage of total sample reads) of the 30 most abundant families in soil.

**
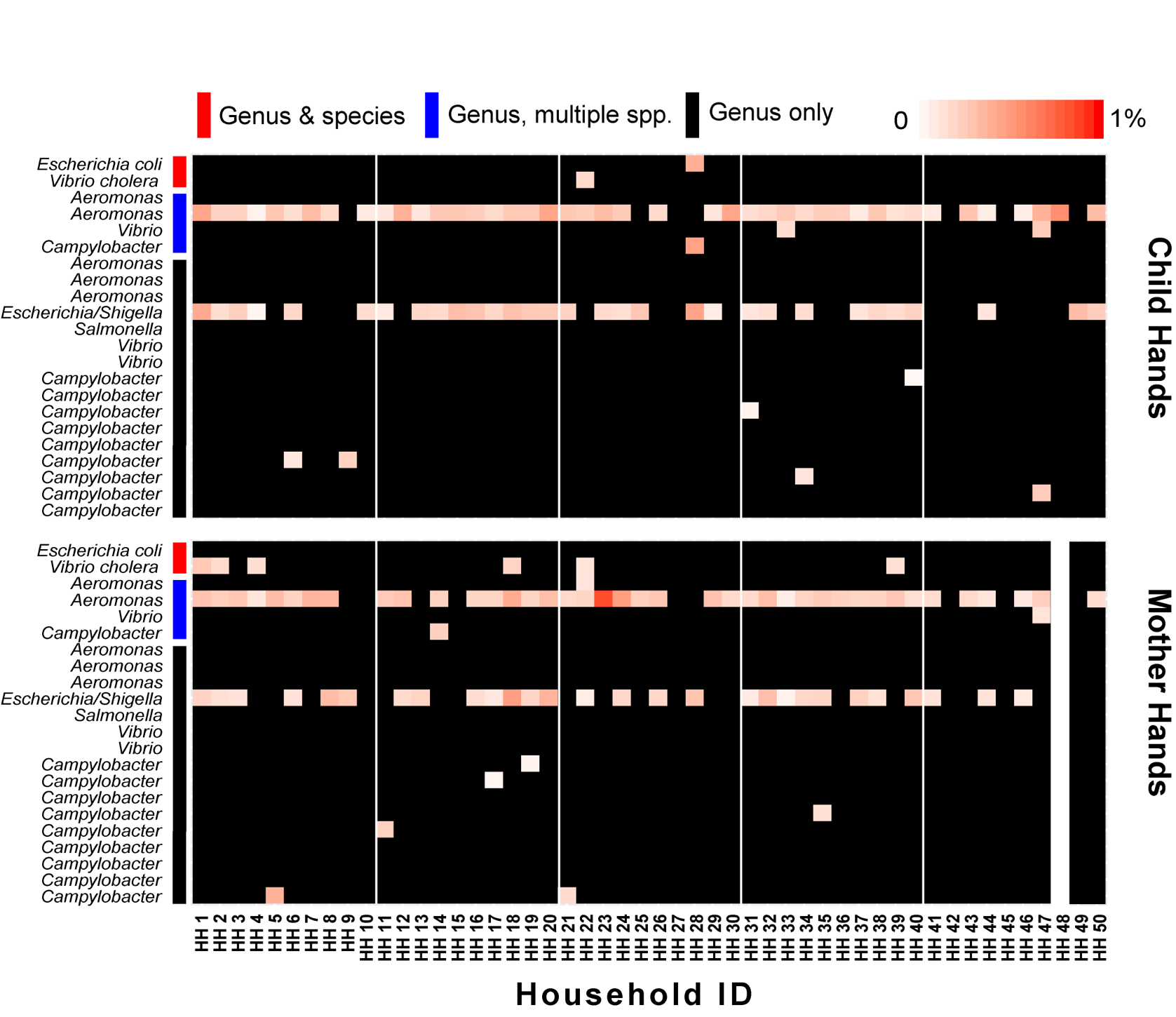
**

**Figure S6**: Relative abundance of ASVs that could be potential pathogens on mother and child hands in 50 households. Note: The mother hand sample in household 48 was omitted due to poor PCR amplification. Identification of pathogens at the genus level is indicated in black, multiple species (one of which could be a pathogen) in blue, and genus and species in red.


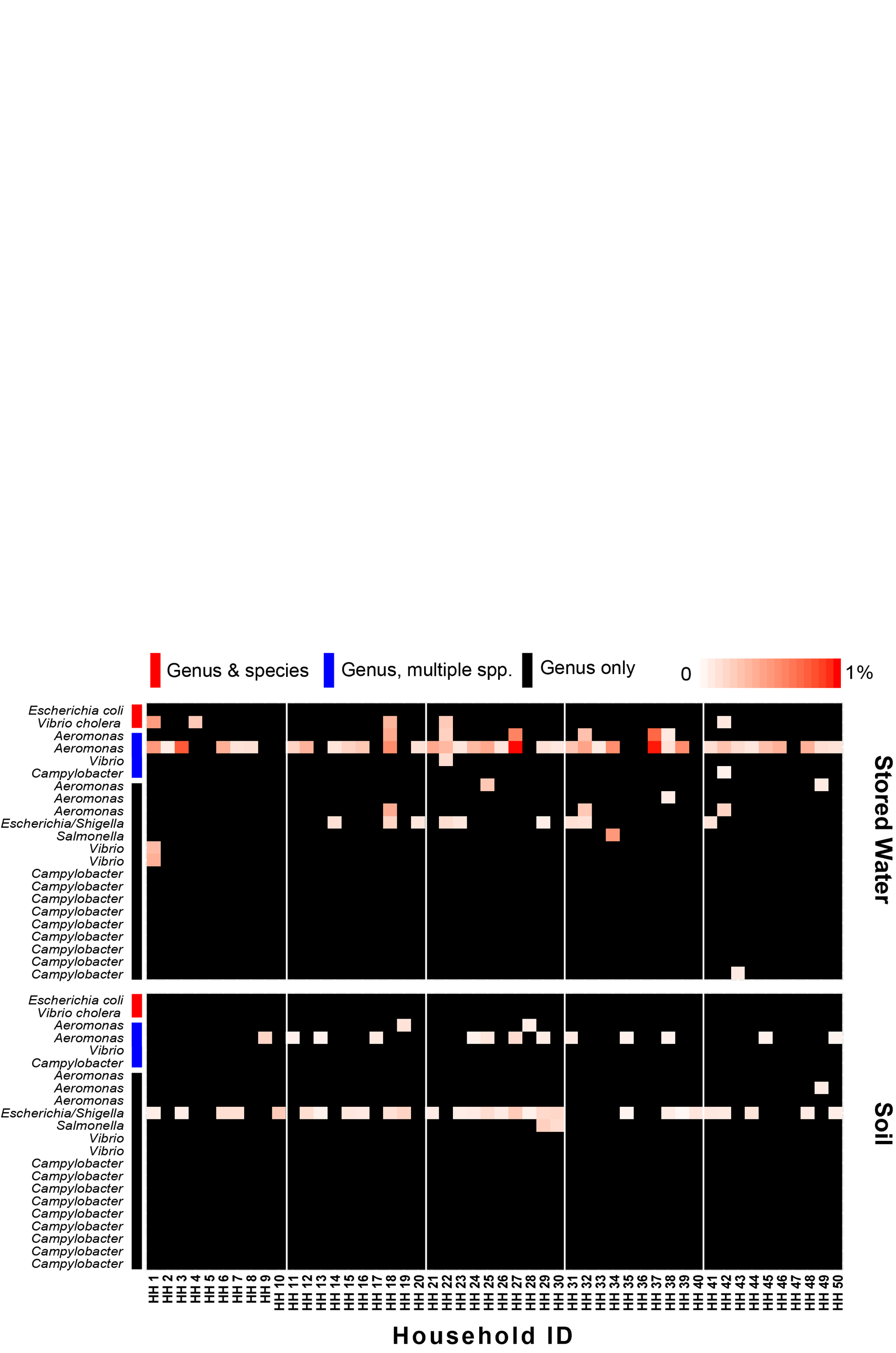


**Figure S7**: Relative abundance of ASVs that could be potential pathogens in soil and stored water in 50 households. Identification of pathogens at the genus level is indicated in black, multiple species (one of which could be a pathogen) in blue, and genus and species in red.
